# Assessment of two POC technologies for CD4 count in Morocco

**DOI:** 10.1186/s12981-020-00289-w

**Published:** 2020-06-10

**Authors:** Elmir Elharti, Halima Abbadi, Rajae Bensghir, Kamal Marhoum El Filali, Hajar Elmrabet, Hicham Oumzil

**Affiliations:** 1 National Reference Laboratory for HIV, Department of Virology, National Institute of Hygiene, Rabat, Morocco; 2 Infectious Diseases Clinic, Ibn Rochd University Hospital Center, Casablanca, Morocco; 3 Medical School and Pharmacy, University Mohammed Vth, Rabat, Morocco

**Keywords:** HIV management, CD4 count, POC, PIMA, FACSPresto, Morocco, HIV monitoring

## Abstract

**Background:**

In the era of “test and treat strategy”, CD4 testing remains an important tool for monitoring HIV-infected individuals. Since conventional methods of CD4 count measurement are costly and cumbersome, POC CD4 counting technique are more affordable and practical for countries with limited resources. Before introducing such methods in Morocco, we decided to assess their reliability.

**Methods:**

In this study 92 blood samples from HIV-infected patients, were tested by PIMA and FACSPresto to derive CD4 count. Flow cytometry using FacsCalibur, was used as reference method for CD4 count comparison. Linear regression, Bland–Altman analysis were performed to assess correlation and agreement between these POC methods and the reference method. In addition, sensitivity and specificity, positive predictive value (PPV), negative predictive value (NPV) and misclassification percentage at 350 and 200 CD4 count thresholds; were also determined. Finally, because FACSPresto can also measure hemoglobin (Hb) concentration, 52 samples were used to compare FACSPresto against an automated hematology analyzer.

**Results:**

The coefficient of determination R^2^ was 0.93 for both methods. Bland–Altman analysis displayed a mean bias of − 32.3 and − 8.1 cells/µl for PIMA and FACSPresto, respectively. Moreover, with a threshold of 350 CD4 count, PIMA displayed a sensitivity, specificity, PPV, NPV, were 88.57%, 94.12%, 91.18%, 92.31%; respectively. FACSPresto showed 88.23%, 96.23%, 93.75% and 92.73%; respectively. Furthermore, the upward misclassification percentage was 8.57 and 5.88%, for PIMA and FACSPresto, respectively; whereas the downward misclassification percentage was 7.84% and 7.54%; respectively. With 200 cells/µl threshold, PIMA had a sensitivity, specificity, PPV and NPV of 83.33%, 98.53%, 93.75% and 95.71%, respectively. Regarding FACSPresto, sensitivity, specificity, PPV and NPV was 82.35%, 98.57%, 88.57% and 95.83%; respectively. Upward misclassification percentage was 5.56% and 5.88%, for PIMA and FACSPresto, respectively; whereas downward misclassification percentage was 4.41% and 4.29%; respectively. Finally, the hemoglobin measurement evaluation displayed an R^2^ of 0.80 and a mean bias of − 0.12 with a LOA between − 1.75 and 1.51.

**Conclusion:**

When compared to the reference method, PIMA and FACSPresto have shown good performance, for CD4 counting. The introduction of such POC technology will speed up the uptake of patients in the continuum of HIV care, in our country.

## Background

Since the advent of HAART therapy, HIV-infected patients have been treated according to CD4 count threshold. Initially the threshold was set to 200 CD4 count, as the question of when to start HAART therapy was not answered [1–3]. However, since 2009, studies have demonstrated the benefit of early initiation of HAART therapy [4, 5] and this treatment threshold was raised to 350 in 2010, and then to 500 CD4 count, in 2013 [6, 7]. Furthermore, in 2014, other studies revealed a benefit of early treatment for all patients, irrespective of their immunologic or virologic status [5, 8]. In fact, it was reported that early treatment is not only beneficial for the HIV-infected individuals themselves, but it can also reduce the viral infectiousness and subsequently the ongoing HIV transmission [8]. This evidence prompted international guidelines to recommend early treatment of HIV-infected individuals. In this framework, in 2015, WHO advocated “Test and treat strategy”. In other words, once a person tested infected with HIV, they should be offered HAART therapy, immediately and regardless of their CD4 count [9]. In this respect, universal access to the treatment has transformed the deadly HIV/AIDS to a chronic disease, in developed countries. In addition, they are also endeavoring to achieve the UNAIDS goal towards ending the epidemic, by 2030 [10–12]. In contrast with this context, the HIV/AIDS remains an important cause of death in resources limited countries, despite significant efforts that aimed at helping these countries access HAART therapy. Thanks to these efforts, 24.5 million patients accessed HAART treatment at the end of 2018 [13]. Nevertheless, 35% of 37.9 million persons living with HIV/AIDS, are still not treated. Consequently, in these settings, access to HAART therapy still prioritized for patients most in need, by using CD4 count [14, 15]. In fact, CD4 count is essential for identifying late presenters that require urgent care, including immediate opportunistic infections treatment, in order to improve their prognosis. Therefore, CD4 count remains an essential tool for HIV management for many low-to-middle income countries, mainly in Sub Saharan African countries, that have adopted “test and treat” approach, but its implementation has been challenging [16–18].

Conventional methods are cumbersome due to manual pipetting and longer incubation periods, but they are expensive, because of a high sample throughput compared to the POC PIMA or FACSPresto, as they can perform up to 32 blood samples per carousel [19]. However, the turnaround times for these results from the central laboratory to the health facility can take from a couple of hours to days to be received by the clinic. Over time though, POC technologies are cheaper than conventional methods because of better clinical outcomes, less clinic visits, no loss of results, no transport costs and time off work are taken into account [20].

Morocco is regarded as low HIV prevalence area, since this prevalence is less than 0.1%, and the current estimate of persons living with HIV/AIDS is around 22,000 [21, 22]. In early 90s the Moroccan ministry of health has developed and implemented a national response to curb the epidemic HIV/AIDS, within the country. The impact of this national response has been further strengthened since the advent of Global Fund in 2003, which helped to scale up HIV prevention treatment and care within the country.

Morocco has followed WHO guidelines for initiating the antiretroviral treatment. Since 2015, “test and treat strategy” has been adopted by the Moroccan ministry of health. Despite the introduction of this strategy, CD4 testing is still required for the management of HIV-infected people, within Morocco. In fact, according to national guidelines, CD4 count is required for all newly HIV-diagnosed patients in order to decide starting and discontinuing the prophylaxis of opportunistic infections, for patient with late presentation. Besides, CD4 count is still measured for patients initiating HAART therapy, and once the HIV viral load is fully suppressed and CD4 count exceeds 350 cells/µl, the monitoring is based only on HIV viral load testing.

POC technologies are being deployed within the country, in order to facilitate the access to CD4 count. Because all HIV management centers are available in regional and some provincial hospitals, with laboratory facilities, it was decided to install this POC CD4 count technology within laboratories of these hospitals. Additionally, within these laboratories, CD4 count is introduced in parallel with HIV viral load technique, in order to make HIV monitoring more accessible, throughout the country. In this framework, we decided to evaluate two POC CD4 count technologies, PIMA and FACSPresto, by comparing them to a reference method.

## Methods

In this study, we have used 92 remnant samples collected within EDTA tubes, regularly addressed for CD4 count monitoring, from HIV management center, for HIV-infected patients, in University Hospital Center Ibn Rochd, in Casablanca, between May 26 and September 4, 2015. Samples were routinely collected and tested in unlinked anonymous manner [23] to determine CD4 count. Ethical approval for this study was obtained from the Ethics committee of Biomedial Research, Medical School and Pharmacy, University Mohammed V^th^, Rabat, Morroco.

### The reference method

Throughout the entire evaluation, the reference method was a single platform flow cytometry with three-color reagent kit, performed on a standard clinical instrument. Samples were stained by mixing 50 µl of whole blood, with 10 µl of CD3FITC/CD4PE/CD45PercP (Beckton-Dickinson), in tubes containing beads (TrueCount, Beckton-Dickinson), and then incubated for 15 min. Samples were lysed and fixed during 15 min, by adding 450 µl of lysing solution (FACS lysing solution, Beckton-Dickinson). All the incubations were performed at room temperature. Percentage and absolute CD4 count were determined on flow cytometer (FacsCalibur, Beckton-Dickinson) by using CellQuest Pro software [24]. All steps were performed according to manufactures’ instructions.

### PIMA technique

PIMA CD4 technology consists of a portable device for CD4 count testing, using disposable cartridge that comprises dried reagents, made of anti-CD3 and anti-CD4 antibodies conjugated to dyes. The cartridge was opened and 25 µl of blood sample were added, then it was capped and loaded into the analyzer. After 20 min of incubation inside the analyzer, the absolute CD4 count was determined when all steps are successful, otherwise an error report is provided, and in this case the sample was repeated once to determine the CD4 count. The results are displayed on the screen device and printed automatically.

### FACSPresto technique

FACSPresto CD4 system is a device that determines CD4 count, by using dried reagent preloaded in disposable cartridges. The reagents are made of anti-CD3, anti-CD4, anti-CD14 and anti-CD45RA antibodies conjugated to fluorescent dyes, as well as an integrated quality control (QC). A volume of 25 µL of blood sample is transferred to the cartridge which is capped and incubated at room temperature, during 18 min. Then, the cartridge is loaded onto the FACSPresto analyzer, and the reading takes around 4 min. Afterwards, the device prints absolute and percentage CD4count, as well as hemoglobin (Hb) concentration in g/dl. In case of reading problem, the analyzer generates an error report, and in this case the sample is repeated once, in an attempt to measure CD4 count. Results are shown on the analyzer screen and printed automatically.

For both POC CD4 count methods, the number of samples per hour was estimated. Furthermore, sensitivity, specificity, positive predictive value (PPV) and negative predictive value (NPV) were calculated for a CD4 count thresholds of 350 and 200 cells/µl, for both techniques. At these thresholds, we have also measured, the upward misclassification percentage, i.e.: number of samples incorrectly identified as above the threshold using POC technique PIMA or FACSPresto**/**number of samples correctly identified as below the threshold using the reference method × 100, and the downward misclassification percentage, i.e.: number of samples incorrectly identified as below the threshold, using POC PIMA or FACSPresto technique**/**number of samples correctly identified as above the threshold using the reference method ×100. Finally, Hb concentration measured by FACSPresto, was compared to Hb determined by an automated hematology analyzer (Coulter Ac.T diff, Beckman Coulter) on 52 samples.

### Quality control

For the reference method, maintenance is performed on daily basis for the cytometer FacsCalibur cytometer. In addition, quarterly maintenance of each 3 months consisted of verifying laser alignment as well as the status of the machine is performed by specialist engineer. Finally, the instrument is calibrated in each run, with beads (Calibrite Beads,Beckton-Dickinson).

Regarding the POC PIMA, there are two quality control cartridges, one with low CD4 count (detection range: 111–231 cells/µl) and another with high CD4count (detection range: 686–1274 cells/µl), which are tested in each run.

For FACSPresto, two types of QC, one for CD4 count and another for Hb measurement, are printed automatically, each time the device is switched on.

In addition, a commercial stabilized blood (Immunotrol Cells, Beckman Coulter) with CD4 count range: 598 ± 165 cells/µl, was used as an internal quality control throughout the study and tested in each run, by the reference method as well as by PIMA and FACSPresto techniques. Moreover, given the fact that FACSPresto can also determine Hb with the same cartridge used for CD4 count; we therefore decided to assess the Hb measured by this technique. As we didn’t have an internal control when we began the study, we used Immunotrol Cells (Beckman Coulter), as an internal control. We ran it 10 times using the reference method, to calculate the mean and the SD (4.40 ± 0.11 g/dl), and then we used it each run, by FACSPresto and the reference method, during the study. The technologists were trained on using the PIMA and FACSPresto techniques as well as on the reverse pipetting. All tests were performed during 24 h after venipuncture, by the same technologist.

### Statistical analysis

The mean of CD4 count, the SD and the % of CV were calculated when necessary. Linear regression was performed to assess the correlation between the two methods of interest. The fitted regression line was presented graphically with the line of perfect agreement (y = x). The coefficient of determination (R^2^) was also calculated. Furthermore, the agreement between these technologies and the reference method was studied by Bland–Altman analysis. In this case, the mean bias as well as the 95% limits of agreement (LOA), i.e. mean bias ± 1.96 SD, were calculated. Finally, for Hb measurement linear regression and Bland–Altman were performed as well to evaluate the results obtained by FACSPresto, using an automated hematology analyzer as a reference technique.

## Result

### Quality control

In the present study, PIMA low control and high control gave a mean ± SD and %CV of 153.27 ± 4.76 and 3.10, 1015.36 ± 8.79 and 0.87; respectively. The internal quality control QC of FACSPresto is automatically performed each time the device is switched on. In addition, within 2 min, the device prints automatically 2 QC ticks, one for CD4 count and the other for Hb, meaning the internal control is passed and the machine is ready for samples measurement.

During this study, the CD4 count of a commercial stabilized blood (with CD4 count target 598 ± 165) tested by the reference method and by PIMA and FACSPresto techniques, was determined (Table 1). Regarding absolute CD4 count, the reference method gave a mean ± SD of 650.89 ± 60.91 and a %CV of 9.3. PIMA and FACSPresto had a mean ± SD and %CV of 686.78 ± 53.22, 7.75 and 633.56 ± 52.57, 8.30; respectively. As far as the CD4 percentage concerned, the reference method and FACSPresto gave a mean ± SD and a %CV of 47.73 ± 2.87, 5.75; 45.18 ± 2.32, 5.13; respectively (data not shown).

**Table 1:** Stabilized blood analyzed by the three methods

	Reference method	PIMA	FACSPresto
Mean	650.89	686.78	633.56
SD	60.91	53.22	52.57
%CV	9.36	7.75	8.30

### Performance of PIMA and FACSPresto

During this study, we tested 92 samples with the reference method and by PIMA and FACSPresto technologies. There were six samples that PIMA instrumentation was unable to read and five no reads by FACSPresto. Table 2 depicts the statistics related to studied samples by all three methods.

**Table 2:** Characteristics of samples studied by the reference method, PIMA and FACSPresto techniques

	Reference method		FACSPresto		PIMA
CD4 count	Absolute count	%	Absolute count	%	Absolute count
Number	92	92	87	87	86
Range	(14–1501)	(1–44)	(27–1481)	(1–45)	(34–1019)
Mean ± SD	466.81 ± 301.26	21.44 ± 10.89	479.63 ± 280.04	22.55 ± 10.56	446.33 ± 236.01
95% CI for the mean	404.43–529.20	19.19–23.70	419.95–539.32	20.30–24.80	395.64–497.01

**Fig. 1:**
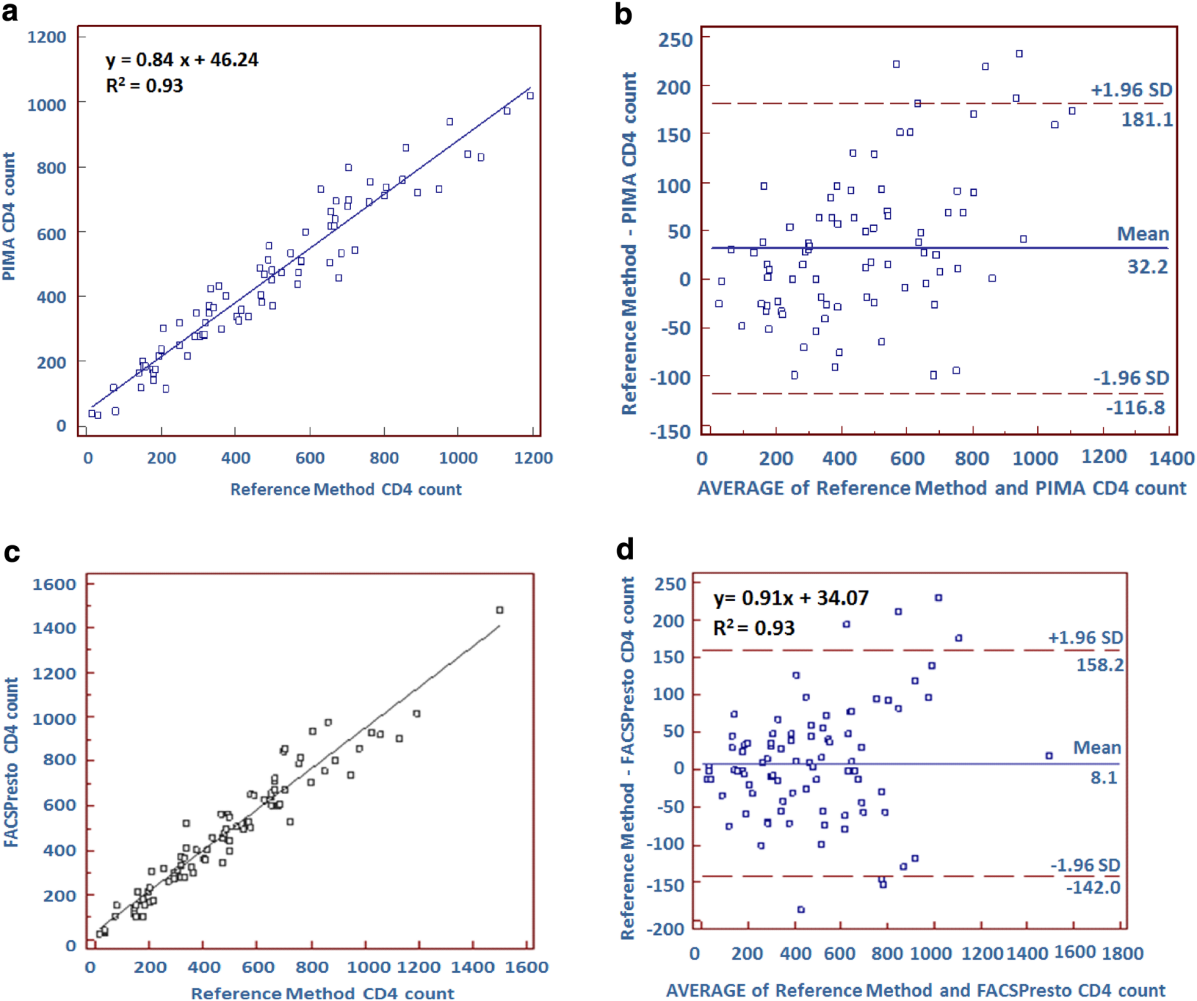

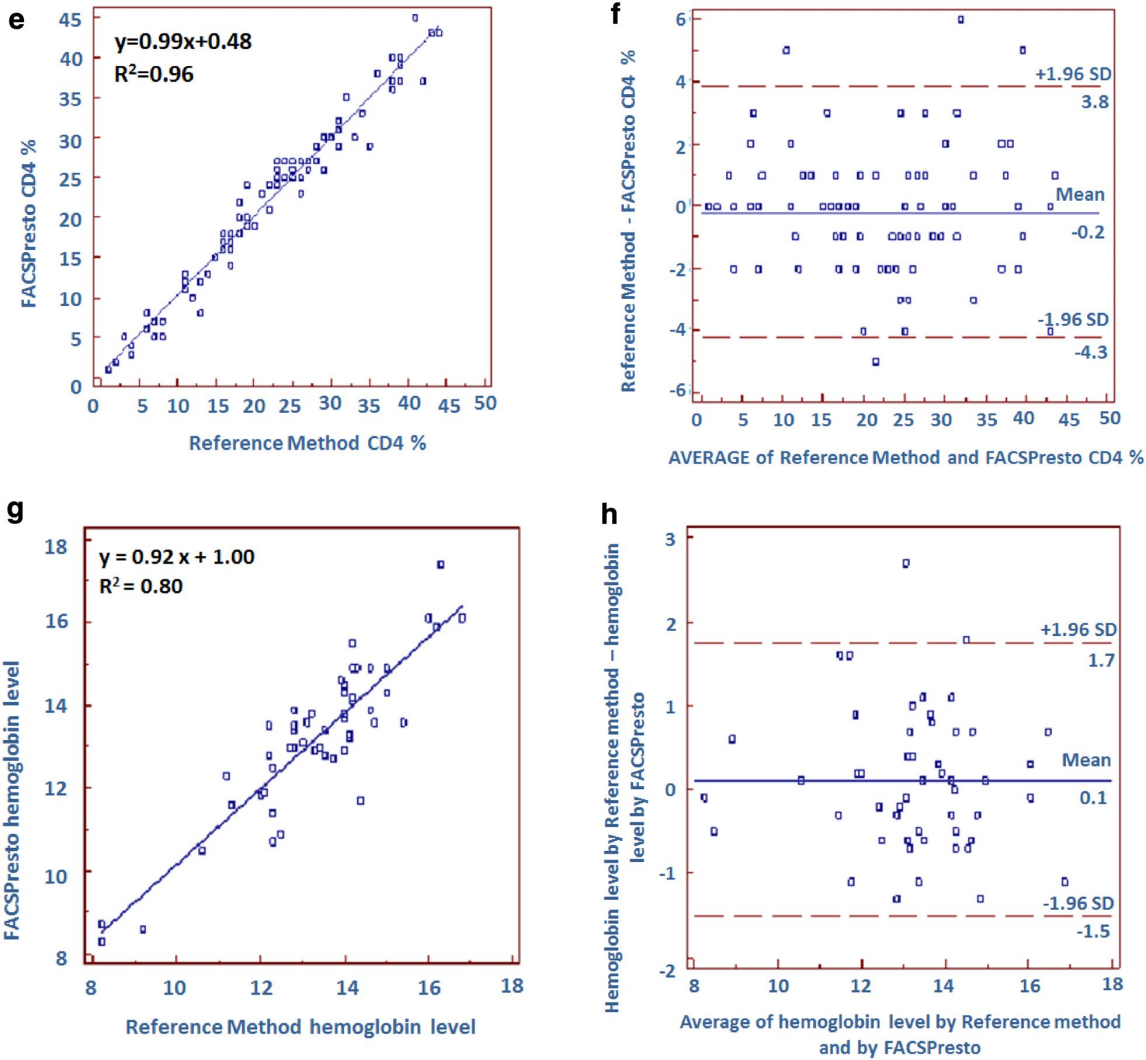
Linear regression and Bland–Altman analysis for comparison between PIMA, FACSPresto techniques and the reference method. Linear regression (**a**) and Bland–Altman analysis (**b**) for absolute CD4 generated by PIMA. Linear regression (**c**) and Bland–Altman analysis (**d**) for absolute CD4and % CD4 (**e**, **f**) determined by FACSPresto. Linear regression (**g**) and Bland–Altman analysis (**h**) for FACSPresto for hemoglobin level. The reference method for CD4 count is the tritest run on FacsCalibur and for hemoglobin is Coulter Ac.T diff

Correlation and agreement assessment between PIMA, FACSPresto and the reference method are represented in Fig. 1. The comparison of CD4 count measurement between PIMA and the reference method, displays a determination coefficient R^2^ = 0.93 and a regression equation y = 0.84x + 46.24. Regarding FACSPresto technology, the comparison gives an R^2^ = 0.93 and regression equation *y* = 0.1x + 34.07, for absolute CD4 count. Concerning the percentage of CD4 count, the regression equation is y = 0.99x + 0.48 and R^2^ = 0.96. Furthermore, the agreement between each technique and the reference method, studied by Bland–Altman analysis, shows a mean bias of − 32.3 cells/µL, with LOA ranging from − 181.3 to 116.8, for PIMA technology. When the testing is performed by FACSPresto method, the mean bias is − 8.1 cells/µL, with LOA varying from − 150.8 to 141.0. The mean bias obtained by FACSPresto is lower than that obtained by PIMA (p = 0.0384, data not shown). The mean bias is − 0.2 with a LOA between − 3.8 and 4.3, for the CD4 count percentage generated by FACSPresto. We have also measured the sensitivity, specificity, PPV, NPV, using thresholds of 350 and 200 CD4 count (Table 3). Regarding 350 CD4 count threshold, PIMA had a sensitivity, specificity, PPV and NPV of 88.57% (73.26–96.80), 94.12% (83.76–98.77), 91.18% (77.40–96.89) and 92.31% (82.46–96.80), respectively. FACSPresto showed a sensitivity, specificity, PPV and NPV of 88.23% (72.55–96.70), 96.23% (87.02–99.54), 93.75% (79.30–98.33) and 92.73% (83.53–96.98), respectively. Furthermore, the upward misclassification percentage was 8.57% (1.80–23.10) and 5.88% (0.70–19.70), for PIMA and FACSPresto, respectively; whereas the downward misclassification percentage was 7.84% (2.20–18.90) and 7.54% (2.10–18.200), for PIMA and FACSPresto, respectively (Table 3).

**Table 3:** Sensitivity, specificity, PPV, and NPV by PIMA and FACSPresto

Technique	CD4 threshold (cells/µl)	Sensitivity (95% CI)	Specificity (95% CI)	PPV (95% CI)	NPV (95%CI)	Upward misclassification (95% CI)	Downward misclassification (95% CI)
PIMA	350	88.57% (73.26–96.80)	94.12% (83.76–98.77)	91.18% (77.40–96.89)	92.31% (82.46–96.80)	8.57% (1.80–23.10)	7.84% (2.20–18.90)
	200	83,33% (58.58–96.42)	98.53% (92.08–99.96)	93.75% (67.95–99.07)	95.71% (88.82–98.43)	5.56% (0.10–27.30)	4.41% (0.90–12.40)
FACSPresto	350	88.23% (72.55–96.70)	96.23% (87.02–99.54)	93.75% (79.30–98.33)	92.73% (83.53–96.98)	5.88% (0.70-19.70)	7.54% (2.10–18.20)
	200	82.35% (56.57–96.20)	98.57% (92.30–99.96)	88.57% (66.39–99.00)	95.83 (89.17–98.47)	5.88% (0.10–28.70)	4.29% (0.90–12.00)

When a threshold of 200 cells/µl was used, PIMA displayed a sensitivity, specificity, PPV and NPV of 83.33% (58.58–96.42), 98.53% (92.08–99.96), 93.75% (67.95–99.07) and 95.71% (88.82–98.43), respectively. With FACSPresto sensitivity, specificity, PPV and NPV were of 82.35% (56.57–96.20), 98.57% (92.30–99.96), 88.57% (66.39–99.00) and 95.83% (89.17–98.47), respectively. Upward misclassification percentage was 5.56% (0.10–27.30) and 5.88% (0.10–28.70), for PIMA and FACSPresto, respectively; whereas the downward misclassification percentage was 4.41% (0.91–12.40) and 4.29% (0.90––12.40), for PIMA and FACSPresto, respectively.

Since FACSPresto can also generate the hemoglobin concentration, we have compared the results of 52 samples tested by FACSPresto to those measured by an automated hematology analyzer. Results (Fig. 1) show an R^2^ = 0.80 and a regression equation, y = 0.92x + 1.00. The mean bias was − 0.12 with a LOA between − 1.75 and 1.51.

Finally, we have assessed the throughput of both CD4 count techniques (data not shown). Regarding PIMA, the number of samples that can be tested per hour is three samples; whereas FACSPresto can perform seven tests an hour.

## Discussion

In the era of the UNAIDS 90 90 90 target aimed at eliminating HIV/AIDS by 2030, cost and complexity of technology still represent the most prohibitive challenges that hamper scaling up HIV tests used to monitor HIV-infected people, in developing countries. This brings about delay or inability to access HIV care. In this context, POC CD4 counting technology, known to be cheaper over time with respect to patient clinical management, over time with respect to patient clinical management; represents an excellent tool to speed up the linkage of HIV-infected individuals to care cascade, and therefore help optimize the management of HIV-infected persons, for these settings. However, the assessment of such techniques is a major prerequisite before their introduction in HIV management. In fact, their performance should be evaluated to avoid tests that generate unreliable results, which may put patients to unnecessary risk of morbidity and mortality, associated with HIV/AIDS [25]. Additionally, the evaluation is crucial to inform and guide decision-making, regarding the appropriate choice of the reliable and affordable techniques [26].

In this study, we have evaluated two POC CD4 count techniques, PIMA and FACSPresto.

PIMA has displayed good performance when compared to the reference method. In fact, we have found a mean of bias of − 32.3 cells/µl. This result concurs with other studies that found a mean bias ranging from − 32 to − 22 cells/µl [27, 28]. Actually, PIMA is a well established technology that has been used for many years, in developing countries, particularly in sub-Saharan Africa, for the measurement of CD4 count, to monitor HIV-infected people [29, 30].

As far as FACSPresto method concerned, this technology performs well when compared to the reference method, since the mean bias is − 8.1 cells/µl and 0.2%, for absolute count and CD4 percentage, respectively. These findings are in line with previous studies that report similar results [31, 32].

The sensitivity, specificity, PPV and NPV of both POC techniques were also evaluated against the reference method at thresholds of 350 and 200 CD4 count. The results displayed a sensitivity and specificity around 90% and PPV and NPV around 90%, for 350 cells/µl threshold, for both methods. With a threshold of 200 cells/µl, sensitivity was around 82% and specificity was around 99%, for both methods. PPV was around 90% and NPV was around 96%, for both methods. Furthermore, upward and downward misclassification percentages were around 8%, with a threshold of 350 cells/µl; for both methods. Moreover, upward and downward misclassification percentages, were around 6% and 4%, respectively; for both methods, with a threshold of 200 cells/µl. With a cut-off of 350 CD4 count, 8% upward misclassification, mean that 8% of patients will not be monitored for CD4 count, even though they have less that 350 CD4; and 8% will be monitored for CD4 count despite having more than 350 cells/µl. Regarding a cut-off de 200 cells/µl, 6% of patients will be misclassified as having more than 200 cells/µl, whereas 4% will be considered as having less than 200 cells/µl. Despite these limitations, POC CD4 count remains a key tool, for monitoring HIV-infected people, in developing countries.

The results of the present study were similar to other studies using these technologies [32, 33] and witness the reliability of CD4 count measurement by PIMA and FACSPresto technologies, for monitoring HIV-infected individuals [28, 34].

It is worthy of note that the device FACSPresto can provide also the CD4 count percentage, which is important for monitoring HIV-infected children who are less than 5 years [35]. Importantly, it has also the advantage to measure the level of hemoglobin, which is essential for a timely management for HIV-infected individuals with hemoglobin lower level, during hematological abnormalities [36]. Such technologies that can perform simultaneous tests with the same reagents and the same instrument are important for developing countries, since they could help providing a rapid testing and optimizing the available resources.

In this study, we have assessed the throughput of both POC CD4 count techniques. Regarding PIMA, the number of samples that can be tested per hour is three samples; whereas FACSPresto can perform up to seven tests an hour. Therefore, if we can assume that, the working time per day is 8 h; PIMA can analyze up to 24 samples a day, while FACSPresto can measure up to 56 samples. This difference in the daily throughput is due to the fact that the incubation (around 18 min) takes place within the machine, for PIMA. Consequently, the time between consecutive samples is always 20 min. On the contrary, for FACSPresto, the incubation of samples occurred outside the analyzer (18 min), and each sample reading takes around 5 min.

The rate of no read errors by both methods is 5% for PIMA and 6% for FACSPresto. All samples that lead to this reading failure have les 100 CD4 count, except for one. This rate failure was already reported and can exceed 10% [37, 38] and then might represent a limitation for these technologies, at least for some late presenters. However, given the affordability, rapidity, and the simplicity of these technologies; they are all-important for developing countries, particularly in low-to-middle income countries, where access to care remains difficult. In these setting, the use of these technologies is crucial for the identification of the HIV-infected late presenters, and therefore essential to save lives.

In this regard, POC CD4 count methods have been used on finger prick blood, in basic healthcare center, in order to speed up the continuum of care of HIV-infected persons. However, the performance of such technology on finger prick seems to be lower than that of venous blood. In this regard, trainings on using these technologies as well as the way of collecting capillary blood are essential for reliable results [38].

The decentralization process of HIV management in Morocco is based on the creation of HIV/AIDS management activity in hospitals as well as the deployment of the HIV tests including diagnosis tests, viral load and CD4 count testing, in the hospital laboratory. The main goal of such decentralization is to provide an immediate linkage to care and therefore strengthen a timely management of HIV-infected people, which is primordial for the UNAIDS three 90s goal.

In our context, these 2 techniques were evaluated on venous blood, since CD4 count is planned to be deployed in parallel with HIV viral load, within clinical laboratories of hospitals where HIV management is available.

These POC technologies are user-friendly; however trainings represent a perquisite to ensure reliable results and therefore enhance a timely HIV management.

Limitations: FACSPresto has the advantage to generate also the percentage CD4 count which is important for children; nevertheless, in our study, this technique was evaluated only on adults’ samples. Moreover, these POC techniques were assessed using venous blood; however, we didn’t evaluate them on finger-prick sample.

## Conclusion

In this study, we have demonstrated that both technologies PIMA and FACSPresto can be used to generate reliable CD4 count among HIV-infected patients. These methods can enhance the linkage to care for HIV-infected persons, in Morocco and probably in other developing countries.

## Data Availability

The data generated or analyzed during the current study are available from the corresponding author on reasonable request
